# Sequential dependencies in recognition memory are decision based

**DOI:** 10.1177/17470218251317122

**Published:** 2025-02-12

**Authors:** Michelle A Dollois, Chris M Fiacconi

**Affiliations:** Department of Psychology, University of Guelph, Guelph, Ontario, Canada

**Keywords:** Recognition memory, sequential dependencies, autocorrelation, motor priming

## Abstract

Decision perseveration is consistently observed in recognition tests, such that judgements tend to repeat (e.g., “old” responses tend to follow “old” responses) across trials. This effect has been found across a range of testing styles, including old/new judgements, judgements of frequency, and confidence, and has been interpreted as reflecting the transfer of mnemonic information between trials. However, an alternative explanation that response repetition is rather the product of motor action perseveration has not yet been fully evaluated. Despite the range of response styles used across studies, repeat decisions have consistently been confounded with repeat motor responses. Across three experiments, the present study divorces decision repetition from motor priming, to determine whether decision perseveration maintains. Experiments 1 and 2 found that when participants switch hands between trials, decisions are still more likely to repeat than switch. Similarly, Experiment 3 found no difference in the influence of Previous Decision when mouse paths were able to repeat between trials compared with when they could not. In addition, all experiments show a speed advantage for repeating decisions that cannot be attributed to motor priming. We conclude that decision carryover during recognition tests is ultimately a decision-based effect. The results are discussed in terms of mnemonic models of information transfer.

There is now a wealth of research showing that information that is nominally irrelevant to a memory decision can shape memory judgements (e.g., processing fluency, Jacoby & Dallas, 1981; Jacoby & Whitehouse, 1989; Rajaram, 1993; Whittlesea, 1993; Whittlesea et al., 1990). A subset of this work has specifically focused on understanding the phenomenon of sequential dependencies in recognition decisions (Annis & Malmberg, 2013; Dollois et al., 2024; Duncan et al., 2012; Düzel & Heinze, 2002; Kantner et al., 2019; Malmberg & Annis, 2012; Patil & Duncan, 2018; Ratcliff & Starns, 2009). Studies from this area have observed that decisions made during a recognition test are not made independently, but are in part determined by features of the previous trial and judgement. These sequential dependencies appear to sway decisions as much as 15% (Dollois et al., 2024; Malmberg & Annis, 2012). Despite their substantial contribution to memory decisions, this area is relatively understudied. Although sequential dependencies are consistently observed, their underlying sources are still unsettled, thus warranting further attention. The present study addresses this issue by investigating a potential motoric explanation.

The most frequently reported sequential dependency in recognition tests is a positive dependency between decisions. Across many different testing procedures—old/new recognition tests (Annis & Malmberg, 2013; Dollois et al., 2024; Malmberg & Annis, 2012; Ratcliff & Starns, 2009), judgements of frequency (Annis & Malmberg, 2013; Malmberg & Annis, 2012), and confidence ratings (Kantner et al., 2019; Ratcliff & Starns, 2009)—it is dependably observed that responses are more likely to repeat than change between trials. Within the context of a traditional old/new recognition test, this manifests as “old” decisions being more probable following an “old” judgement than a “new” one. Interestingly, this pattern appears regardless of the status (target or lure) of the test probes on either trial (Dollois et al., 2024; Malmberg & Annis, 2012). This suggests that there is a carryover of the subjective experience of an item, its mnemonic impression, which is incorporated into the next decision.

Much of the research into sequential dependencies in recognition has been inspired by the well-documented sequential dependencies in perceptual decisions (see Manassi et al., 2023; and Pascucci et al., 2023, for reviews). As such, the proposed mechanisms for response carryover during recognition memory tests have often paralleled those described in the perception literature. Within the perception literature, serial dependencies between trials are sometimes cast as a consequence of our visual system skewing present perception to more closely align with recent perception to maintain continuity between moments while navigating a complex visual reality (Pascucci et al., 2023). Mechanistically, this is expressed as either a change in the subjective experience of the perceptual signal to more closely match the recent percept (e.g., Fischer & Whitney, 2014; Holland & Lockhead, 1968; Tong & Dubé, 2022) or a change in decision bias which increases the range of signals that would reproduce the same response (e.g., Treisman & Williams, 1984). Both mechanisms produce equivalent behaviour in the form of sequential dependencies. By framing this process in the terms of signal detection theory (SDT; Green & Swets, 1966; Macmillan & Creelman, 2004) with references to either changes in signal or criterion, it is easy work to extend these explanations into recognition memory. Indeed, Annis and Malmberg’s (2013) attempt to model decision dependencies during recognition tests assumed the blending together of test probe information across trials, taking the approach of a signal change. Similarly, Kantner et al. (2019) modelled carryover in confidence decisions using a “sticky evidence” mechanism that pulled subsequent familiarity signals towards each other under certain conditions. Alternatively, others (Ratcliff & Starns, 2009; Treisman & Williams, 1984) hypothesised a criterion shifting account in which liberal shifts in the old/new decision criterion follow a preceding “old” response. In all cases, these explanations assume that the source of the sequential dependencies is *mnemonic* in nature, rooted in either changes in the familiarity signal or decision processes that underlie retrieval. However, as posited by Dollois et al. (2024), there is another, much less interesting explanation as to why responses tend to repeat across trials: motor priming.

Although the methods used in the studies listed above vary in terms of decision style (old/new judgements, judgements of frequency, confidence ratings), they typically share a consistent response mapping between trials. This means that to make a repeat decision (e.g., “old”) participants would also make a repeat motor action (e.g., press “1” on the keyboard). Thus, it is possible that the positive response dependencies observed across studies is not the product of mnemonic processes but rather comes from a motoric source. If there were a prepotent tendency to repeat motor actions, this would result in the illusion of decisions repeating across tests so long as the mapping between decision and motor action remained constant.

Indeed, in the action planning literature, history effects are common. One example is a response onset benefit for repeating the same motor action in consecutive trials (Randerath et al., 2015; Seegelke et al., 2021; Valyear et al., 2019; Valyear & Frey, 2014, 2015). This is typically measured by varying the effector between actions, such that for some response pairs the action is carried out by the same hand, while for other trials the hand switches between actions. When the same hand is used, the time taken to initiate a response is consistently faster than when hands change (Seegelke et al., 2021; Valyear & Frey, 2014, 2015). Seegelke et al. (2021) went so far as to manipulate effector type (hand/foot), body side (left/right), and movement direction (inward/outward) within event pairs. They found that trials with the most overlap (e.g., hand—left—outward) between priming and probe events showed the greatest speed benefit for responding. This speed advantage is often discussed in terms of efficiency, where the reuse of a recently executed action plan is less costly than forming and executing a new one (Randerath et al., 2015). This is corroborated with brain imaging research, which has found repetition suppression in sensorimotor control areas when actions are repeated (Valyear & Frey, 2015). It is possible that this benefit is also present when making responses during a memory test and perhaps alters response choices.

The influence of action plan efficiency is worth considering because there is a relationship between the action onset speed benefit and action choice probability. Valyear et al. (2019) found that not only were responses faster when hand use repeated, but participants were more likely to use the same hand they had previously used to complete another action even when that choice was more biomechanically costly (i.e., a further reach path). Moreover, they are not the only researchers to establish that actions perseverate between trials. Both Dixon et al. (2012) and Glover and Dixon (2013) found that when participants were asked to grasp novel objects designed to allow two possible grip orientations, grip choice tended to repeat between objects. Even after accounting for the potential influence of visual feedback, it was found that motor priming was the most compelling explanation (Glover & Dixon, 2013). Similarly, reach trajectories are also influenced by recent experience (Cohen & Rosenbaum, 2004; Jax & Rosenbaum, 2007; van der Wel et al., 2007). Jax and Rosenbaum (2007) observed that if participants first had to reach around an obstacle to access a target, the reach path on the subsequent trial, even without an obstacle present, would show a more curved path, partially repeating the previous motor experience. Taken together, there is a considerable amount of evidence that motor priming is able to influence not only the speed of response onset but also the probability of response choice. This calls into question whether the response perseveration observed in recognition decisions is merely the product of motor priming.

An important difference between motor priming in the studies described above and what is observed in memory studies is that in the motor paradigms there is no conflict with the primed action. When choosing between two viable grips, repeating a motor choice will not disadvantage the participant, nor will making a slight detour in reach path on the way to a target. However, in a memory test, to make the same motor response may be at odds with the desired memory decision. It is currently unclear whether this is sufficiently distinct to eliminate the impact of motor priming. If not, in the absence of motor repetition, decision-based sequential dependencies may be significantly weakened. There are hints that the response carryover effect can occur in the absence of motor priming (Kantner et al., 2019; Experiment 1), but as of yet there are no studies that have attempted to directly measure its impact or establish the magnitude of its contribution.

There do, however, appear to be other forms of autocorrelation between recognition trials that cannot be explained by a motor priming account. Dollois et al. (2024) recently demonstrated that previous trial content, in addition to previous decision, influences recognition judgements. They observed that both the perceptual (orthographic) and conceptual (semantic) similarity between consecutive trials was predictive of responding, such that as similarity increased so too did the probability of making an “old” judgement. This effect sometimes runs counter to decision perseveration (e.g., similar consecutive items increase the odds of an “old” response even after a “new” response) and therefore cannot be explained through motor priming alone. However, it has not been established whether response- and content-based dependencies are produced by the same mechanism. Therefore, testing the influence of motor action repetition on memory decisions is an important step in understanding sequential dependencies in recognition tests.

## Current study

The current study aims to disentangle motor repetition from decision repetition in recognition testing. To accomplish this, we have orthogonally manipulated decision and motor response in three experiments. By doing so, we can create four critical conditions: when motor response and decision repeat, when motor response repeats but decision does not, when motor response does not repeat but decision does, and when neither motor response nor decision repeat. With this approach, we can isolate the contribution of motor priming to the decision process and determine whether there is a tendency to repeat decisions even when the motor action is not repeated. If we continue to observe decision perseveration even on trials when motor actions are not repeated, this will support an account that assumes a mnemonic source for sequential dependencies in recognition decisions.

Within this study, we are defining *motor priming* as any time an exact motor action can be repeated in subsequent trials. This term is being contrasted against *decision* which refers to the sorting of test probes into “old” (studied) and “new” (not studied) categories. In addition, although the discussion of content carryover in recognition tests is interesting, this study focuses solely on sequential dependencies between memory decisions, as they are more directly confounded with motor perseveration.

## Experiment 1

The aim of Experiment 1 was to conduct a conceptual replication and extension of Experiment 2a from Dollois et al. (2024). The methods were largely kept consistent with the addition of one factor to the design that allowed us to examine the role of motor priming. Namely, we had participants make some recognition decisions with their left hand, and some with their right. With this design, we could now ask whether making the same judgement (i.e., “old” or “new”) hinged on executing the same motor action (i.e., using the same hand). If the response effect that has been observed across many studies (Annis & Malmberg, 2013; Dollois et al., 2024; Kantner et al., 2019; Malmberg & Annis, 2012; Ratcliff & Starns, 2009) is in fact an example of motor priming, then decisions should be less likely to repeat when it requires a different motor response (switching hands) compared with a repeated motor response (repeating hands). In addition, if motor priming is at play, it may differentially increase the reaction time (RT) to make a repeat decision. In other words, making the same decision (e.g., an “old” decision followed by another “old” decision) should be faster when the same hand is used than when hands change. This hypothesis is based on work that has shown the speed benefit for repeating actions predominantly hinges on whether the same effector is used (Seegelke et al., 2021; Valyear et al., 2019; Valyear & Frey, 2014, 2015). Although these studies use action onset as the measure of speed, we predict that with faster onset, total RT will also vary. It should be noted that although the methods used below include the same letter set manipulation employed by Dollois et al. (2024), we have collapsed across Content conditions for the purposes of this article as our primary concern is with the Response carryover effect.

### Methods

All experiments reported in this study were approved by the Research Ethics Boards (REB) at the University of Guelph, under REB #17-08-027. All experiments were conducted online, and only proceeded after participants gave written informed consent.

#### Participants

All participants were undergraduate students from the University of Guelph, who completed the study in exchange for credit towards courses. Participants were pre-screened such that only those who had normal or corrected-to-normal vision had self-reported English proficiency, were right-handed, and were self-reported “fluid” typists were eligible to participate. Participants were excluded from the final samples if they reported using memory aids for the test (e.g., writing items down during study), if they displayed patterned responding, or if they had poor performance on the multiple-choice style quiz that tested knowledge of the experiment instructions. Patterned responding was defined as making less than two “old” or “new” judgements across the entire test. Poor quiz performance was defined as getting less than 50% of quiz answers correct on the first try. Following the experiment, participants were asked to estimate the proportion of the test for which they kept their fingers placed on the instructed keys. Responses were made via multiple-choice selection (“0-20% / almost none of the test,” “20-40% / some of the test,” “40-60% / about half of the test,” “60-80% / most of the test,” “80-100% / almost all of the test”). Only participants who reported keeping their fingers in place for the majority of the experiment (more than 60% of the time) were included in the results. This was done to ensure that analysed participants were changing hands between trials and experiencing a motoric benefit by using the same fingers when intended.

A power analysis was conducted based on the magnitude of the response carryover effect reported in Dollois et al. (2024; Experiment 2a). The BUCSS (Anderson & Kelley, 2020) package in R software (R Core Team, 2024) was used to account for publication bias and assurance. This analysis directed that we should have a minimum sample of 40 participants to achieve 0.99 assurance and power. Our final sample exceeded this prescription to account for the interaction effects we were seeking.

Of the 72 participants who successfully completed Experiment 1, 2 participants were removed for reporting the use of memory aids during the test, 16 were removed due to poor performance on the instruction quiz, and 6 participants were removed for not keeping their hands in place for the majority of the task. The mean performance on the quiz in the final sample was *M* = 88.89% correct on the first try (*SD* = 15.88%). After exclusions, the final sample included 48 participants. Within the final sample, the mean age was *M* = 19.27 (*SD* = 1.77, range = 18–28), with 37 participants identifying as women and 11 as men.

#### Materials

The experiment was created in *PsychoPy* (version 2020.1.3; Peirce et al., 2022) and hosted online by Pavlovia.org (2023). All participants completed the study online. Three hundred 5-letter pronounceable nonword stimuli were pulled from the ARC nonword database (Rastle et al., 2002) ensuring that none were English pseudohomophones. Identical to the methods of Dollois et al. (2024; Experiment 2a), stimuli were formed using two distinct letter sets, such that items could be divided into two groups with no shared letters between them. As this factor was collapsed across for all analyses below, we refrain from describing this in more detail; however, it should be noted that participants were not made explicitly aware of the letter set manipulation, and previous research has shown that this manipulation is sufficiently subtle to go largely unnoticed (Dollois et al., 2022; Fiacconi et al., 2020). For a more detailed account of these methods and a list of items, see Dollois et al. (2024).

#### Procedure

The experiment consisted of a study, distractor, and test phase. The item list at study was comprised of 150 items presented in a random order. During study, participants were presented with the items one at a time and were required to type each one. Items were presented centralised on the screen, and typed letters were echoed to the screen below the presented item. Study time was self-paced defined by the amount the time it took to type and submit each item (by typing “enter”). A 500 ms fixation cross separated each study trial. Prior to beginning the study phase, participants were made aware of the upcoming test and its format and completed practice trials to familiarise themselves with the typing procedure (as many trials as it took to successfully type two sequential items correctly).

Following the study phase, participants completed 5 min of arithmetic questions which acted as a distractor task to diminish recency effects at test. This was immediately followed by the test phase. During test, all studied items and an equal number of lures (150 additional items, 300 trials total) were displayed individually, centralised on the screen. To progress through the trials, participants made an old/new judgement for each item. Recognition decisions were self-paced, with a 500 ms fixation cross separating each trial.

To manipulate the influence of motor priming during test, trials either required a response to be made with the left or the right hand. On any given test trial, the words “OLD” and “NEW” would appear on either the right or left of the central test probe. With the presentation of the decision labels, the letter of the appropriate response key was also shown. If appearing on the left, the letters Q and X were presented with the decision options (e.g., “Q: OLD”). If appearing on the right, the letters M and P were shown. Participants were instructed to keep both hands placed on the keyboard with one finger per response key throughout the test. Regardless of the hand used to respond, each response was exclusively associated with the upper or lower keys (e.g., to make an “old” response participants would only ever use the Q and P keys), counterbalanced across participants. Trials were randomised such that an equal number of trials required responses with each hand, and that there was a near equal number of trial pairs that required using the same hand and changing hands. Due to there being an odd number of trial pairs, there would either be one less repeat-hand pair or change-hand pair for each participant.

With these methods, we can break down responses by two primary factors, Previous Decision (old, new), which refers to the judgement of an item’s status, and Response Hand (repeat, switch), which refers to whether the same hand was used to make the response relative to the previous trial. Response Hand indicates the potential presence of motor priming, such that when hands repeat the same motor action may be primed. In addition, RT analyses use an alternative factor, Decision Repetition (repeat, switch), which captures whether the previous old/new judgement matched the current judgement.

#### Data availability statement

All experiment and analysis materials have been made available on the Open Science Framework (https://osf.io/ex7nb/).

### Results

All reported analyses were conducted using R software. Effect size measures are reported alongside traditional inferential statistics tests with their respective 95% confidence intervals (CIs). When repeated-measures comparisons are being reported and visualised, CIs follow Morey’s (2008) method of correcting for within-subject comparisons. Prior to analyses, responses with extreme RTs were trimmed from the data. This included all responses faster than 200 ms, and any which exceeded 2.5 standard deviations from each participant’s mean RT. This was done to ensure that responses included in the analyses reflected intentional decisions. On average, RT trimming removed 4.47% of each participant’s trials.

Participants were reliably able to differentiate between old and new items at test. The mean hit rate across participants was *M* = 0.57, 95% CI = [0.55, 0.59], and mean false alarm rate was *M* = 0.47, 95% CI = [0.45, 0.49]. These rates produced a mean *d*′ = 0.27, 95% CI = [0.19, 0.35]. Participants showed an overall neutral decision criterion, *C* = −0.06, 95% CI = [−0.18, 0.07]. These response patterns are consistent with past research using similar methods (Dollois et al., 2024). For raw hit and false alarm rates broken down by condition, see Table 1.

**Table 1.** Raw hit and false alarm rates for Experiment 1.

		Hits		False alarms	
		Mean	95% CI	Mean	95% CI
Overall		0.57	[0.55, 0.59]	0.47	[0.45, 0.49]
Previous response	Response hand				
Old	Repeat	0.63	[0.59, 0.66]	0.53	[0.49, 0.57]
	Switch	0.59	[0.55, 0.62]	0.49	[0.46, 0.53]
New	Repeat	0.54	[0.50, 0.58]	0.43	[0.39, 0.46]
	Switch	0.55	[0.52, 0.59]	0.43	[0.39, 0.46]

**Figure 1.**
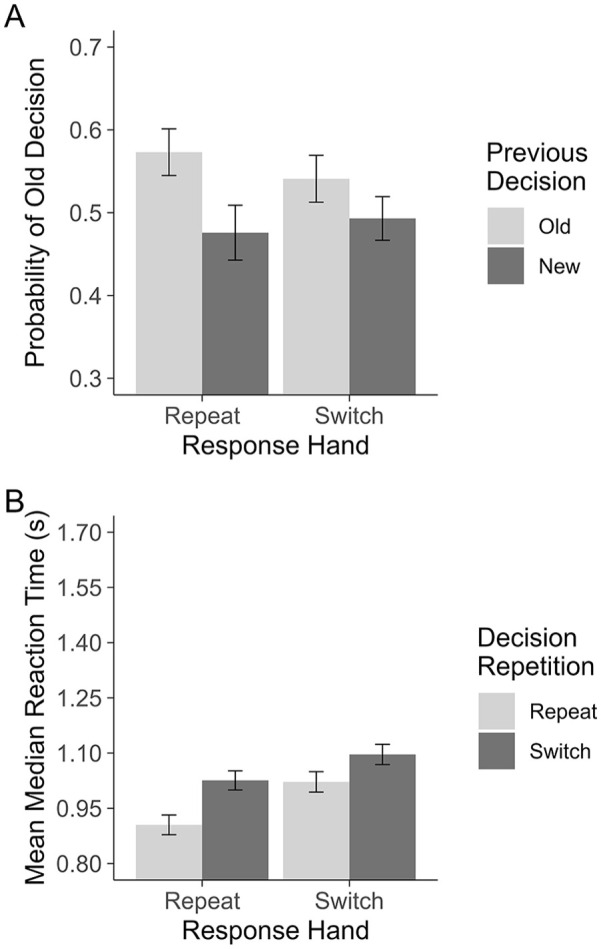
Experiment 1: Plot A shows the probability of making an “old” decision on a given trial. Plot B shows the RTs for repeating and switching between recognition decisions when motor responses are primed (Response Hand: Repeat) and unprimed (Response Hand: Switch). All error bars are based on Morey (2008), corrected for within-subjects comparisons.

Primary to our question of whether the commonly found decision carryover effect is merely the product of motor priming, we compared the conditional probability of responding “old” following “old” and “new” responses when the response hand repeated or switched from the previous trial. To test this, we conducted a 2 (Previous Decision: old, new) × 2 (Response Hand: repeat, switch) repeated-measures analysis of variance (ANOVA). As the effect of Previous Decision has been found to be robust in past research, this effect will be evaluated with a one-tailed test. This revealed a main effect of Previous Decision, *F*(1,47) = 13.49, one-tailed *p* < .001, $\eta_{p}^{2}$ = .22, and no main effect of Response Hand, *F*(1,47) = 0.51, *p* = .480, $\eta_{p}^{2}$ = .01. The main effect of Previous Decision reflects the increased probability of making an “old” decision following an “old” relative to a “new” judgement. However, more critical to the question of motor priming, this test also revealed an interaction, *F*(1,47) = 4.73, *p* = .035, $\eta_{p}^{2}$ = .09, indicating that the magnitude of the decision carryover was moderated by whether the same hand was used. To determine whether changing hands eliminated the decision carryover effect, a paired *t*-test was conducted on only the trials for which participants changed hands. This test still revealed a small effect of Previous Decision on “old” responding, *t*(47) = 2.27, *p* = .028; however, the magnitude of this effect is smaller than when hands repeated, switch: *d* = 0.29, 95% CI = [0.03, .54], versus repeat: *d* = 0.63, 95% CI = [0.29, 0.96]; see Figure 1. This result indicates that even in the absence of motor priming, as defined by switching hands, an “old” decision on the previous trial was still more likely to lead to another “old” decision relative to when the previous decision was “new.”

Consistent with Dollois et al. (2024), the decision carryover effect was characterised by different decision biases following “old” and “new” decisions, without an accompanying difference in sensitivity. Collapsing across motor conditions, decisions were made against a more liberal criterion following an “old” judgement, *C* = −0.17, 95% CI = [−0.25, −0.09], than a “new” judgement, *C* = 0.05, 95% CI = [−0.03, 0.13], *t*(47) = 3.99, one-tailed *p* < .001, *d* = 0.53, 95% CI = [0.24, 0.80]. No such difference was found in *d*′: following an “old” decision, *d*′ = 0.24, 95% CI = [0.17, 0.31], following a “new” decision, *d*′ = 0.32, 95% CI = [0.25, 0.39], *t*(47) = 1.70, *p* = .095, *d* = 0.26, 95% CI = [−0.05, 0.57].

If motor priming is contributing to repeat decisions, we would expect repeat decisions made with the same hand to be faster than changed decisions. In addition, this speed benefit should only be seen when hands repeat. To test this, a 2 (Decision Repetition: repeat, switch) × 2 (Response Hand: repeat, switch) repeated-measures ANOVA was conducted on mean median RTs. The ANOVA revealed a main effect of Decision Repetition, *F*(1,47) = 39.61, *p* < .001, $\eta_{p}^{2}$ = .46, and a main effect of Response Hand, *F*(1,47) = 51.94, *p* < .001, $\eta_{p}^{2}$ = .52. The effect of Response Hand reflects an overall increase of speed when using the same hand in consecutive trials, and the main effect of Decision Repetition reflects an overall increase in speed when making the same decision in consecutive trials. More importantly, both main effects were qualified by a significant interaction, *F*(1,47) = 4.09, *p* = .049, $\eta_{p}^{2}$ = .08. To unpack this interaction, a paired *t*-test was conducted on only the trials for which participants changed hands to confirm whether the RT effect had been eliminated. This test still found a small but significant effect, *t*(47) = 3.75, *p* < .001; however, the magnitude of this effect is smaller than when hands repeat, switch: *d* = 0.30, 95% CI = [0.13, 0.47], versus repeat: *d* = 0.52, 95% CI = [0.33, 0.71], see Figure 1. This result indicates that even when motor priming was prevented, there was still a consistent speed benefit for making the same decision in consecutive trials.

### Discussion

Experiment 1 attempted to selectively eliminate motor priming for half of the test trials by having participants change response hands between some trials. Compatible with a motor priming explanation for decision carryover, we found both an effect of Response Hand (i.e., motor priming) on the carryover effect and an effect of Response Hand condition on the RT benefit for repeat decisions. These results imply that when a motor action can repeat, the probability of making an “old” response following another “old” response relative to a “new” response increases, thus facilitating the response carryover. Similarly, the speed benefit for repeating a decision is also enhanced when the same motor action is being used. However, both of these interactions are qualified by the simple main effects within the Switch Hand condition. Although the perseveration effect and RT benefit were larger when motor priming was permitted, when hands switched between trials both effects persisted.

We see two potential explanations for these results. The first is that we successfully eliminated motor priming in the Switch Hand condition, thus removing the contribution of motor priming from the results. This would suggest that even in the absence of motor priming there is still a small effect of previous decision on recognition judgements. In addition, this would suggest that making repeat decisions, even without making repeat motor responses, is faster than changing decisions (this idea will be further expanded upon in “General Discussion”). Both results suggest that information is crossing between trials at test to influence the decision process. Alternatively, it may be the case that we did not successfully eliminate motor priming in the Switch Hand condition, and the remaining simple main effects are the product of reduced but still present priming. As the global mapping of “old” and “new” responses was constant between hands (e.g., “old” was always the upper key and “new” was always the lower key on the keyboard, regardless of hand), it is possible that participants benefitted from a more general priming of the response direction which was applied to both hands on each trial. If this were the case, this would produce the effects reported above. Indeed, multiple action perseveration studies have found that perseveration that is maintained between hands (Dixon et al., 2012; van der Wel et al., 2007). Although in these studies the target of the motor action remained constant (e.g., reaching for the same object), it is possible that action repetition also appears when the target changes but maintains certain features (i.e., pressing a different but similarly located key). The remaining two experiments attempt to further separate the motorically primed and unprimed conditions to resolve this debate.

## Experiment 2

To address the possibility that Experiment 1 did not successfully remove motor priming as intended, Experiment 2 followed the same general methodology and analysis approach but with less motor overlap between trials that allow for motor priming and those that don’t. Experiment 2 will still operationalise motor priming by comparing trials when response hand repeated and switched, but the mapping of old and new responses for each hand will no longer be mirrored. We reason that this should prevent a more global map of responses to be primed for both hands simultaneously because, for example, “press the upper key for old” will no longer be applicable for both hands. This is bolstered by research in the bimanual action literature, which has found that both hands can seemingly share an action plan when they are symmetrical, but show substantial cost when they are not, even if quite similar. Albert and Ivry (2009) observed that when drawing shapes with both hands simultaneously, responses were fast and accurate when both hands drew the same shape. However, when one of the shapes was rotated 90 degrees, performance fell. This suggests that even with a small adjustment, the compatibility between response mappings may be reduced.

If the results of Experiment 1 are indicative of a partial elimination of motor priming in the Switch Hand condition, then we would expect the interactions between Response Hand and both Previous Decision on “old” responding and Decision Repetition on RT to be larger here. In other words, if response carryover is entirely due to motor priming, we should see no simple effect of Previous Decision on “old” responding and no simple effect of Decision Repetition on RT when response hand changes between trials.

### Methods

#### Participants

As in Experiment 1, all participants were undergraduate students from the University of Guelph, who completed the study in exchange for credit towards courses. Pre-screening and exclusions were identical to Experiment 1 with the addition of one more restriction. In addition to restricting the analyses to participants who kept their hands placed on the instructed keys, we also considered whether the asymmetrical response mapping used may lead to more response errors. To address this, we asked participants for what percentage of the test they accidentally made the wrong response on account of hand switching. Responses were made via multiple-choice selection (“0-20% / almost none of the test,” “20-40% / some of the test,” “40-60% / about half of the test,” “60-80% / most of the test,” “80-100% / almost all of the test”). Only participants who reported that the majority of trials were not mistakes (less than 40%) were included for analysis.

Of the 104 participants who successfully completed Experiment 2, 20 were removed due to poor performance on the instruction quiz, 11 participants were removed for not keeping their hands in place, and 20 were removed for high response errors due to hand switching. The mean performance on the quiz in the final sample was *M* = 96.23% correct on the first try (*SD* = 10.66%). After exclusions, the final sample included 53 participants. Within the final sample, the mean age of the group was *M* = 19.47 (*SD* = 2.07, range = 18–31), with 43 participants identifying as women, 9 as men, and 1 as gender non-binary.

#### Materials

Materials were identical to Experiment 1.

#### Procedure

Procedure was almost entirely identical to Experiment 1. The only difference was in how participants responded with each hand during the test phase. Similar to Experiment 1, to manipulate the influence of motor priming during test, trials either required a response to be made with the left or right hand. On any given test trial, the words “OLD” and “NEW” would appear either to the right or left of the central test probe along with the letter of the key to press to make that response. The critical change for Experiment 2 is that one hand had the old and new responses aligned vertically, using the I and M (right) or W and Z (left) keys, while the other hand had the responses aligned horizontally, using the J and L (right) or A and D (left) keys. Which hand would have which alignment was randomised between participants, as were the specific positions within each alignment assigned to old and new responses. For example, a participant may have used I to respond “old” and M to respond “new” on right-hand trials and used A to respond “old” and D to respond “new” on left-hand trials. Trials were randomised such that an equal number required responses with each hand, and that there was a near equal number of trial pairs that required using the same hand and changing hands.

### Results

The same RT trimming procedure used in Experiment 1 was applied to this experiment. On average, RT trimming removed 2.76% of each participant’s trials.

Participants were reliably able to differentiate between old and new items at test. Mean hit rate across participants was *M* = 0.59, 95% CI = [0.57, 0.60], and mean false alarm rate was *M* = 0.45, 95% CI = [0.43, 0.46]. These rates produced a mean *d*′ = 0.36, 95% CI = [0.31, 0.42]. Again, participants showed an overall neutral decision criterion, *C* = −0.04, 95% CI = [−0.14, 0.05]. For raw hit and false alarm rates broken down by condition, see Table 2.

**Table 2.** Raw hit and false alarm rates for Experiment 2.

		Hits		False alarms	
		Mean	95% CI	Mean	95% CI
Overall		0.59	[0.57, 0.60]	0.45	[0.43, 0.46]
Previous response	Response hand				
Old	Repeat	0.64	[0.62, 0.67]	0.50	[0.47, 0.53]
	Switch	0.62	[0.59, 0.64]	0.48	[0.45, 0.51]
New	Repeat	0.56	[0.53, 0.59]	0.40	[0.37, 0.43]
	Switch	0.52	[0.49, 0.55]	0.41	[0.38, 0.43]

To determine the contribution of motor priming towards decision carryover, we compared the conditional probability of responding “old” following “old” and “new” responses when the response hand repeated or switched from the previous trial using a 2 (Previous Decision: old, new) × 2 (Response Hand: repeat, switch) repeated-measures ANOVA. This revealed only main effect of Previous Decision, *F*(1,52) = 32.03, one-tailed *p* < .001, $\eta_{p}^{2}$ = .38. The main effect of Response Hand, *F*(1,52) = 3.09, *p* = .085, $\eta_{p}^{2}$ = .06, and the interaction between Previous Decision and Response Hand, *F*(1,52) < 0.01, *p* = .975, $\eta_{p}^{2}$ < .01, did not reach significance.

**Figure 2.**
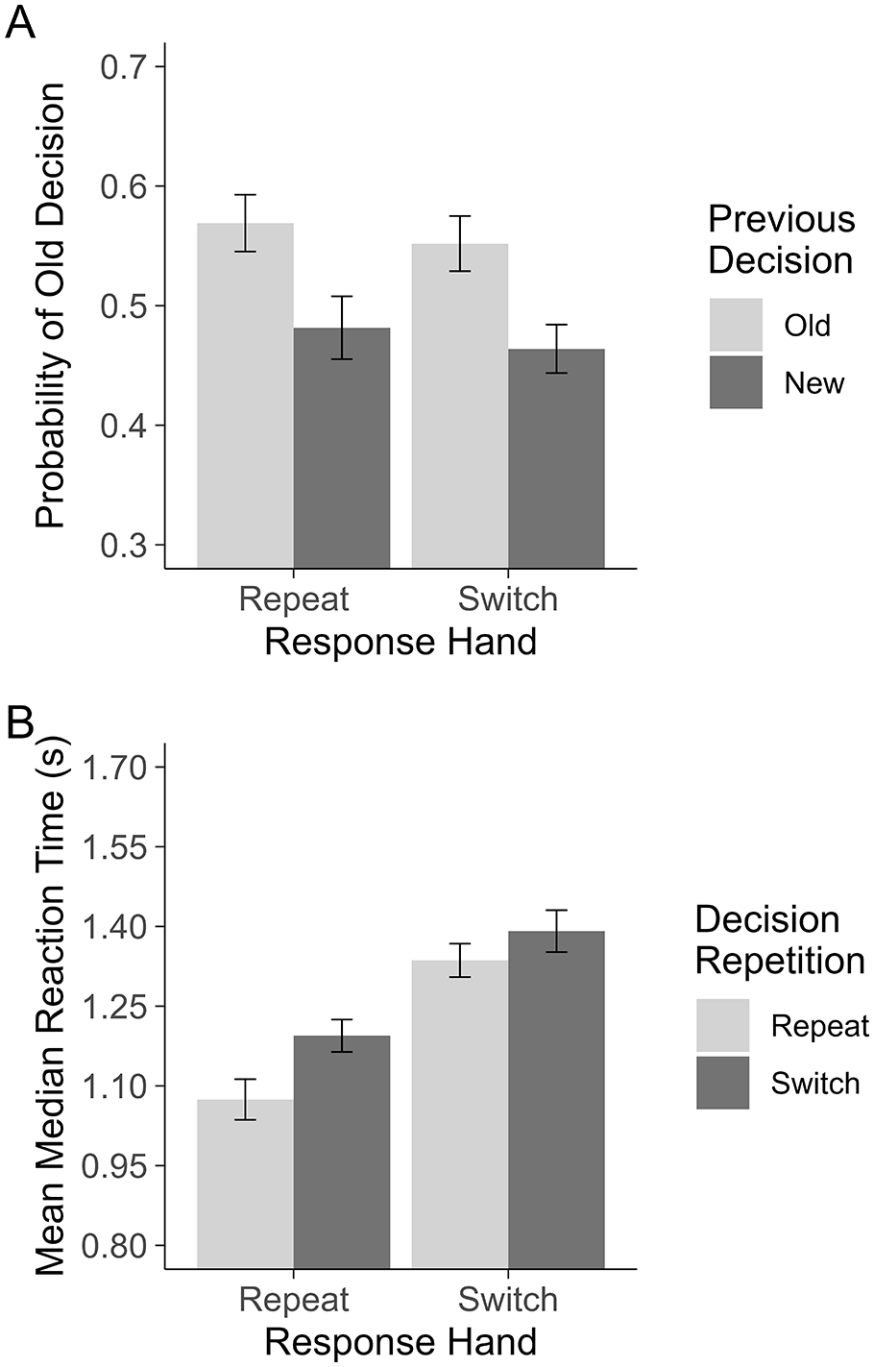
Experiment 2. Plot A shows the probability of making an “old” decision on a given trial. Plot B shows the RTs for repeating and switching between recognition decisions when motor responses are primed (Response Hand: Repeat) and unprimed (Response Hand: Switch). All error bars are based on Morey (2008), corrected for within-subjects comparisons.

As the interaction was central to our research question, we calculated the Bayes factor (BF) for the interaction. Critically, this approach captures the relative likelihood of the alternative versus the null hypothesis given the data. *BF*_10_ values > 1 indicate support for the alternative hypothesis with benchmarks as follows: 1–3 indicates anecdotal (insufficient) evidence, 3–10 moderate evidence, and <10 strong evidence. *BF*_10_ values < 1 indicate support for the null hypothesis with benchmarks as follows: 0.33–1 indicates anecdotal (insufficient) evidence, 0.1–0.33 moderate evidence, and <0.1 strong evidence. In using this method, we can establish whether the observed null result is more consistent with the null hypothesis being true or an underpowered, inconclusive comparison. This test was conducted with the *anovaBF* function from the *BayesFactor* package (Morey & Rouder, 2024) in R using the default Cauchy priors and treating Participant as a random factor. This test revealed that there was moderate evidence in support for the null hypothesis, *BF*_10_ = 0.20. This indicates that the null interaction likely reflects that the effect of Previous Decision does not depend on Response Hand. Unlike Experiment 1, these tests reflect that “old” decisions were more probable following an “old” decision relative to a “new” decision on the previous trial, and that motor priming played a negligible role in this. See Figure 2 for a visualisation of the means.

As with the previous experiment, the response carryover effect was characterised by a more liberal decision bias following “old” decisions than “new” and no reliable difference in sensitivity. Mean *d*′ following “old” decisions, *d*′ = 0.37, 95% CI = [0.32, 0.43], and following “new” decisions, *d*′ = 0.35, 95% CI = [0.30, 0.41], *t*(52) = 0.51, *p* = .615, *d* = 0.08, 95% CI = [−0.23, 0.39]. Mean *C* following “old” decisions, *C* = −0.16, 95% CI = [−0.21, −0.10], and following “new” decisions, *C* = 0.07, 95% CI = [0.01, 013], *t*(52) = 5.54, one-tailed *p* < .001, *d* = 0.67, 95% CI = [0.40, 0.94].

To corroborate the absence of a motor priming effect in this sample, we again compared the RT benefit of repeating and switching decisions in consecutive trials when using the same or different hands for each response. The 2 (Decision Repetition: repeat, switch) × 2 (Response Hand: repeat, switch) repeated-measures ANOVA on mean median RTs revealed a main effect of Decision Repetition, *F*(1,52) = 18.70, *p* < .001, $\eta_{p}^{2}$ = .27, and a main effect of Response Hand, *F*(1,52) = 152.34, *p* < .001, $\eta_{p}^{2}$ = .75. As in Experiment 1, we found a significant interaction between Decision Repetition and Response Hand, *F*(1,52) = 6.48, *p* = .014, $\eta_{p}^{2}$ = .11. A paired *t*-test was used to test the simple main effect of Decision Repetition on trials when the response hand switched. This test still found a small but reliable effect, *t*(52) = 2.24, *p* = .029; however, the magnitude of this effect is smaller than when hands repeat, switch: *d* = 0.18, 95% CI = [0.02, 0.33], versus repeat: *d* = 0.47, 95% CI = [0.27, 0.67] (see Figure 2). This result indicates that even when motor priming was prevented, there was still a consistent speed benefit for making the same decision on consecutive trials.

### Discussion

Experiment 2 aimed to improve upon the design of Experiment 1 by reducing the amount of response mapping overlap in the two Response Hand conditions. Despite this, not only did we not see the loss of the simple main effect of Previous Decision on “old” responding when response hand switched, we also no longer observed an interaction between Previous Decision and Response Hand. These results suggest that motor priming does not meaningfully contribute to decision carryover between trials.

The above result may appear at odds with the interaction between Decision Repetition and Response Hand on RT; however, we find two considerations that provide cohesion. First, as in Experiment 1, despite the interaction on RT, there remains a simple main effect of Decision Repetition when the response hand changes between trials such that responses are faster when decisions repeat even if hands change. This suggests a fluency or priming associated with the *decision* which is separate from the *response*. This provides additional evidence that decision repetition is not facilitated solely by motor priming. Second, the presence of the interaction confirms that the Repeat Hand condition involves more motor priming than the Switch Hand condition, and yet we see no difference between these conditions when examining decision carryover. This provides evidence that motor priming may facilitate faster repeat responses without increasing the probability of making repeat responses.

In Experiment 3, we attempted a conceptual replication of the previous two experiments to confirm which carryover pattern of results is more reliable. In addition, we again strived to further differentiate the primed and unprimed motor conditions. Although Experiment 2 changed the response mapping, such that “old” decisions did not share exact action features between hands, it is still conceivable, though perhaps unlikely, that we were not successful in fully eliminating motor priming. Consider that in bimanual actions, practice can generate improvement when simultaneously drawing incongruent shapes with each hand (Albert & Ivry, 2009), and that if the two incongruent shapes being drawn can be conceptualised as a whole object, performance improves (Franz et al., 2001). These studies suggest that different motor actions completed by different hands can potentially share an action plan under the right circumstances. Perhaps over the course of the recognition, test participants are able to cohesively represent “old” responses as being both “up” and “left” together. Experiment 3 changes response modalities to remove this concern.

## Experiment 3

In Experiment 3, we expanded upon the previous two experiments by further distinguishing the two motor priming conditions from each other. To accomplish this, we changed from keyboard responses to mouse responses and increased the unpredictability of which motor action will be needed to make a given response. This is an improvement on the previous design because in Experiment 2 the consistent response mapping may have allowed both potential repeat decisions to be primed. If both “old” (or “new”) responses were grouped into a single response representation, then both actions may have been primed together. In Experiment 3, we change the response mappings such that making the same decision the next trial may require the same action, but it is equally likely to require one of two other responses. In addition, if the response location changes, the two alternative responses have been pitted in opposition to each other, such that to prepare for one is to impede the other. This further reduces the benefit of priming any motor action other than an exact repeat because multiple conflicting alternatives are probable. In taking this approach, we can be confident that we have eliminated motor priming on the intended trials.

### Methods

#### Participants

Participants primarily consisted of undergraduate students from the University of Guelph, who completed the study in exchange for credit towards courses. Five participants were removed due to technical error that impacted the collection of demographic information. These students were replaced with paid participants collected through Prolific.com (2024), an online, international participant pool. Pre-screening was identical to Experiments 1 and 2. Exclusion criteria were largely the same as the previous experiments except that self-reports of hand placements and mistakes were not measured and thus not used.

Of the 101 participants who successfully completed Experiment 3, 20 were removed due to poor performance on the instruction quiz, and 2 were removed for reporting the use of memory aids, leaving a final sample of 79. The mean performance on the quiz in the final sample was *M* = 90.72% correct on the first try (*SD* = 15.04%). Within the final sample, the mean age of the group was *M* = 19.46 (*SD* = 2.83, range = 18–35), with 65 participants identifying as women, 13 as men, and 1 as gender non-binary.

#### Materials

Materials were identical to those used in Experiments 1 and 2.

#### Procedure

**Figure 3.**
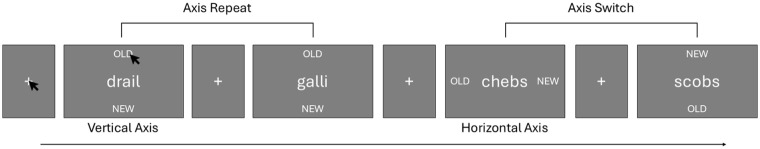
Schematic of Experiment 3 procedure. To progress through the test, participants first clicked on the fixation cross to begin the next trial, and then clicked on either the “OLD” or “NEW” label to finish a trial, as depicted for the first trial above. Decision labels either appeared along the vertical or horizontal axes centred on the test item. If the axis repeated between consecutive trials, the locations of the two decision labels within that axis remained constant. If the axis switched between consecutive trials, the locations of the two decision labels within the new axis was random. Although the width of fixation and item displays vary above, they were equal in the experiment.

The procedure used in Experiment 3 was conceptually the same as in the prior experiments. The study phase was identical; however, the mode of responding differed for the test phase. Instead of making responses using the keyboard, participants used the mouse to select their judgement. On each trial, the test probe was presented centrally in the screen along with the two response labels. The words “OLD” and “NEW” were displayed either above and below the probe or flanking it on either side. Participants responded by using their mouse to click on the label that matched the status of the test item (i.e., they would click on “OLD” if they felt the item was studied, and “NEW” if they felt the item was not studied). On each trial, the axis along which the old and new labels appeared may change or repeat. When the axis repeated (e.g., OLD and NEW appeared above and below the test item on consecutive trials), the respective locations of the two labels along the axis remained constant. When the axis changed between consecutive trials (e.g., OLD and NEW appeared above and below the test item on one trial, and then appeared to the left and right of the item on the subsequent trial), which label appeared at each location along the axis was random (refer to Figure 3 for a depiction of the procedure). With this method, on trials for which the response axis repeated, making a repeat decision required making a repeat motor response, while on trials for which the response axis changed, making a repeat decision could not involve making a repeat motor response. In addition, by randomising the location of the labels when the axis changed, and by placing the potential response targets in opposite directions, it is unclear how any motor priming could still occur on these axis-change trials.

Trials were randomised such that an equal number required responses along each axis, and that there was a near equal number of trial pairs that required using the same axis and changing axes. Due to there being an odd number of trial pairs, there was either one less repeat-axis pair or change-axis pair for each participant.

After making their response, the test item and response labels would disappear, to be replaced by a central fixation cross. To ensure that mouse paths were of consistent lengths across trials and conditions, after selecting a recognition response, participants had to click on the fixation cross to begin the next trial. This ensured that all four possible response locations were equidistant at the beginning of each trial.

### Results

The same RT trimming procedure used in Experiments 1 and 2 was applied to this sample. On average, RT trimming removed 2.94% of each participant’s trials.

Participants were reliably able to differentiate between old and new items at test. Comparable to the previous experiments, mean hit rate across participants was *M* = 0.59, 95% CI = [0.58, 0.60], and the mean false alarm rate was *M* = 0.50, 95% CI = [0.49, 0.51]. These rates produced a mean *d*′ = 0.26, 95% CI = [0.22, 0.29]. Participants showed a liberal decision criterion, *C* = −0.12, 95% CI = [−0.17, −0.06], indicative of being predisposed to making “old” responses regardless of item status. For raw hit and false alarm rates broken down by condition, see Table 3.

**Table 3.** Raw hit and false alarm rates for Experiment 3.

		Hits		False alarms	
		Mean	95% CI	Mean	95% CI
Overall		0.59	[0.58, 0.60]	0.50	[0.49, 0.51]
Previous response	Response axis				
Old	Repeat	0.62	[0.59, 0.64]	0.50	[0.47, 0.53]
	Switch	0.60	[0.58, 0.62]	0.50	[0.48, 0.52]
New	Repeat	0.58	[0.55, 0.61]	0.49	[0.46, 0.53]
	Switch	0.57	[0.55, 0.59]	0.49	[0.47, 0.51]

**Figure 4.**
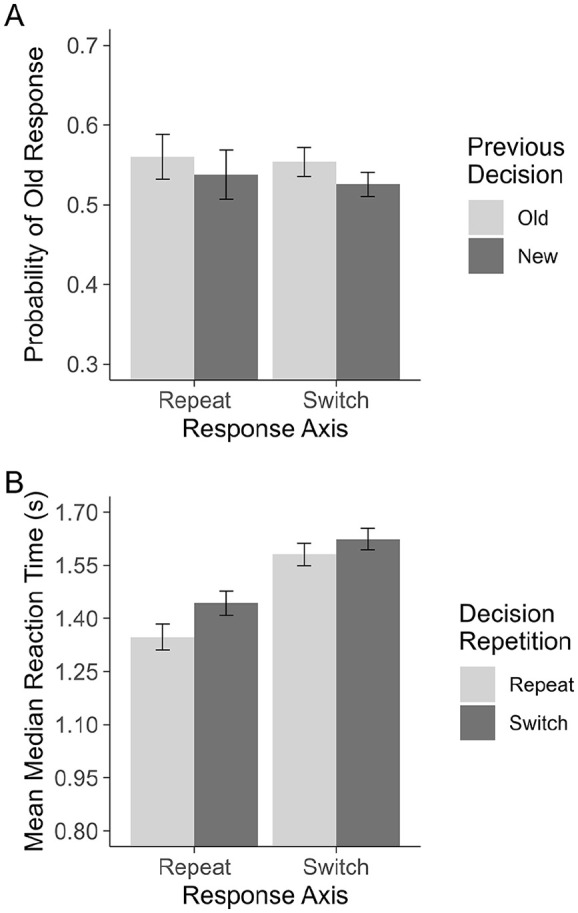
Experiment 3: Plot A shows the probability of making an “old” decision on a given trial. Plot B shows the RTs for repeating and switching between recognition decisions when motor responses are primed (Response Axis: Repeat) and unprimed (Response Axis: Switch). All error bars are based on Morey (2008), corrected for within-subjects comparisons.

Again, we compared the conditional probability of responding “old” following “old” and “new” decisions when the response was motorically primed by the previous trial using a 2 (Previous Decision: old, new) × 2 (Response Axis: repeat, switch) repeated-measures ANOVA. This test revealed only a main effect of Previous Decision, *F*(1,78) = 2.90, one-tailed *p* = .046, $\eta_{p}^{2}$ = .04. The main effect of Response Axis, *F*(1,78) = 1.73, *p* = .193, $\eta_{p}^{2}$ = .02, and the interaction between Previous Decision and Response Axis, *F*(1,78) = 0.05, *p* = .825, $\eta_{p}^{2}$ < .01, did not reach significance. As with the prior experiment, since the interaction effect was central to our research question, we calculated a BF to contextualise the null result. We found that there was moderate evidence in support for the null hypothesis, *BF*_10_ = 0.17, indicating that null interaction effect likely reflects that the effect of Previous Decision does not depend on the response axis repeating. Indeed, although the overall effect of Previous Decision is smaller in Experiment 3 compared with 1 and 2, when examining the simple main effect of Previous Decision on trials without motor priming, a two-tailed *t*-test confirmed an effect, *t*(78) = 2.25, *p* = .027, *d* = 0.27, 95% CI = [0.03, 0.51]. Taken together, these tests reflect that “old” responses were more probable following an “old” response relative to a “new” response on the previous trial regardless of motor priming (see Figure 4).

As with the previous experiments, we tested whether the decision carryover effect was characterised by a change in decision bias. Again, we failed to find a difference in sensitivity following each decision type; following “old,” *d*′ = 0.28, 95% CI = [0.23, 0.34], following “new,” *d*′ = 0.22, 95% CI = [0.17, 0.28], *t*(78) = 1.65, *p* = .103, *d* = 0.25, 95% CI = [−0.05, 0.56]. Unlike in the previous experiments, we were not able to find a reliable difference between decision biases in the current sample; following “old,” *C* = −0.14, 95% CI = [−0.19, −0.09], following “new,” *C* = −0.09, 95% CI = [−0.14, −0.04], *t*(78) = 1.31, one-tailed *p* = .097, *d* = 0.17, 95% CI = [−0.09, 0.42]. However, the pattern of means is consistent with previous experiments and literature.

Finally, we conducted a 2 (Decision Repetition: repeat, switch) × 2 (Response Axis: repeat, switch) repeated-measures ANOVA on mean median RT to investigate whether, as in the previous two experiments, there is a RT benefit when decisions repeat even without repeating motor actions. One participant was excluded from this analysis due to not having observations in every cell. This test revealed a main effect of Decision Repetition, *F*(1,77) = 11.90, *p* = .001, $\eta_{p}^{2}$ = .13, and a main effect of Response Axis, *F*(1,77) = 151.04, *p* < .001, $\eta_{p}^{2}$ = .66. As in the previous experiments, we found a significant interaction between Decision Repetition and Response Axis, *F*(1,77) = 4.81, *p* = .031, $\eta_{p}^{2}$ = .06. A paired *t*-test looking for the simple main effect of Decision Repetition on trials when the axis switched revealed a slight effect, *t*(77) = 2.01, *p* = .048, with an effect size three times smaller than when the axis repeated, switch: *d* = 0.09, 95% CI = [0.00, 0.18], versus repeat: *d* = 0.26, 95% CI = [0.12, 0.40]. A paired-samples Bayesian *t*-test confirmed the inconclusive nature of the simple main effect, *BF*_10_ = 0.84 (see Figure 3).

### Discussion

Experiment 3 mostly replicated the results of Experiment 2. This was most clear when testing the conditional probability of making an “old” response on a given trial: an effect of Previous Decision was observed which did not interact with Response Axis. This not only provides evidence that even in the absence of motor priming decision carryover still occurs, but that the magnitude of the effect is not moderated by switching response axes.

Although the simple main effect of Decision Repetition on RT for Switch Axis trials was minimal, the overall pattern of means was consistent with the previous two experiments. Indeed, when the data of all three experiments were combined, there was strong evidence of no interaction between Experiment and Decision Repetition on RT in the unprimed motor condition, *F*(2,176) = 0.52, *p* = .598, $\eta_{p}^{2}$ = .01, *BF*_10_ = 0.09. It can be noted that the magnitude of the RT benefit for the primed motor condition in Experiment 3 is considerably smaller than in the prior experiments. We suspect that this is a consequence of having participants click on the central fixation cross between each trial. The intervening motor action (returning the cursor to centre screen) likely dampened the strength of the priming for repeating the same mouse path when the next trial began. In addition, it can be noted that RTs were generally slower in this experiment compared with the previous two (mean median RT for Experiment 1 = 1.01 s, Experiment 2 = 1.25 s, Experiment 3 = 1.50 s). We assume that this increase in RT is directly related to the increase in complexity of making a response. Experiment 3 required additional orienting to the location of the response labels before executing a response which likely slowed participants down. The differences between RTs for each experiment maps on neatly to the increased complexity brought on by methodological changes. Regardless, these RT results are secondary to the impact of motor priming on conditional probability when considering our primary research question.

Similar to the pattern of RT results, the overall magnitude of the decision carryover effect was much smaller in Experiment 3 than in Experiments 1 and 2. Again, we suspect this is the product of the methodological changes between experiments. In addition to a change in the mode of responding (mouse click as compared with keypress), the unpredictability of *how* to make an appropriate response on a given trial may have injected additional noise into the decision process. For example, it may be possible that at the start of each trial, the first thing a participant does is orient themselves to the locations of the OLD and NEW response labels, and only after this do they consider the test item and their decision. This attentional detour may weaken the contribution of the decisions from the previous trial, thus shrinking the carryover effect. Most importantly, however, the magnitude of the decision carryover effect did not vary between Response Axis conditions. This is inconsistent with a motor priming account, and instead supports an explanation where *decision* information is crossing between trials.

## General discussion

Across three experiments, we have very clearly replicated the decision carryover effect reported elsewhere in the field (Annis & Malmberg, 2013; Dollois et al., 2024; Kantner et al., 2019; Malmberg & Annis, 2012; Ratcliff & Starns, 2009)—across all experimental conditions we have shown that decisions tend to repeat rather than change relative to the preceding trial. Novel to this study, we have shown that this decision dependency does not hinge on whether repeating decisions co-occur with repeat motor actions. In two of the three experiments reported here, there were no signs of the decision carryover effect being moderated by motor priming. Conversely, in Experiment 1, we observed that the degree to which the current decision reflected the previous decision was dependent on whether the same hand was used in the subsequent trials. However, even in the Switch Hand condition, there was still reliable evidence that decisions were biased towards the previous trial. Potential explanations for the presence of an interaction in Experiment 1 are discussed below.

Also novel to this study was the comparison of RTs for repeated and switched decisions as a function of motor priming. A consistent pattern was observed across all experiments, namely, that decisions were made more quickly when they matched the previous decision even when motor priming was absent. Although motor priming would predict faster RTs for repeat responses within the same hand, something which can explain the interaction between Repeat Decision and Motor Priming (Response Hand/Axis), it cannot clearly explain how there is still an RT advantage for repeat decisions in the absence of motor priming. Taken together, the results presented here are consistent with the account that decision priming is the product of mnemonic information transfer between trials, and not an artefact of repeat decisions being made through repeat motor actions.

Although the overall pattern of results was quite consistent across experiments, it is worth examining the place where they differed and unpacking potential causes. The interaction between Response Hand and Previous Decision in Experiment 1 somewhat calls into question the complete separation of motor priming from decision priming found in the other experiments. If decision priming was truly unrelated from motor priming, why would we see the effect of Previous Decision diminish in its absence? One potential source is the greater overlap of features between the two motor priming conditions relative to the later two experiments. This may be surprising to consider, given that earlier in this report we put forward that the partial response overlap may allow for motor priming to still be present in our “unprimed” Switch Hand condition; this was used to motivate the subsequent experiments. However, in light of the results of Experiments 2 and 3, it is unlikely that the difference between the priming conditions is owed to differences in the degree of positive motor priming present. Instead, what may underlie the disparity is a *negative* priming effect on the Switch Hand trials which is competing with the positive decision priming effect, resulting in a reduced overall difference.

Response repetition costs are a staple feature of task-switching paradigms. It is well known that making the same response when tasks have changed between trials produces costs as seen in increased response speeds and error rates (for reviews, see Kiesel et al., 2010; Monsell, 2003). It is possible that the partial overlap in response features in Experiment 1 created similar conflict when making repeat decisions with different hands. Under this view, changing hands acts as a task change, and thus, the repeated use of response direction (e.g., “up”) may conflict with the switch between hands. There is additional precedent for this in the motor literature which has found response costs when motor plans partially overlap (Fournier et al., 2010; Fournier & Richardson, 2022; Mattson & Fournier, 2008; Stoet & Hommel, 1999; Wiediger & Fournier, 2008), for example, when plans share a directional component like “left” (conflict between responses made with hand vs foot, Stoet & Hommel, 1999; conflict between manual and verbal responses with semantic overlap, Fournier et al., 2010). This aligns well with the reconfiguration account of task-switching (Kleinsorge, 1999; Kleinsorge & Heuer, 1999; other accounts are also compatible, see Druey, 2014) which assumed that actions are arranged hierarchically, and switches at higher levels in the hierarchy induce switches at the lower levels. Applied to Experiment 1, changes in hand may facilitate changes in response, thus reducing the expected probability of repeating a response when hands change. The larger differences in mapping between left and right hand in Experiment 2 would have prevented such interference—the change in response induced by changing hands was equally compatible with either response option, allowing decision priming to occur unimpeded.

An alternative explanation for the interaction found on the probability of giving an “old” judgement in Experiment 1 is that the quality of our sample may have been lower than in Experiment 2. Although we used the reported proportion of the test that participants kept their hands in place on the keyboard as an exclusion criterion in the first experiment, we were not able to exclude participants based on the frequency of accidental responses due to hand switching confusion; this question was only added in the second experiment. If participants were making accidental responses when switching hands that did not reflect intentional decisions, these may have obscured the decision priming effect. As these mistakes would likely be mostly restricted to the Switch Hand condition, we could expect to see the observed interaction. It is worth noting that this explanation is not mutually exclusive from the partial repetition cost explanation. Indeed, in Experiment 2, it is possible that the additional screening question selectively removed participants who were particularly susceptible to response repetition costs and made their report based on that experience.

With this study, we have affirmed that the response-based sequential dependencies observed in recognition tests are much more likely to be a *decision* effect as opposed to a *motor* effect. Earlier in the article we discussed two potential avenues for mnemonic information from one trial to influence the next: decision repetition could potentially reflect a trial-by-trial change in decision criterion, or a change in the experienced memory signal associated with the item. Both accounts are consistent with the RT patterns observed in the present work. It is generally accepted within the SDT model of memory that response speed is inversely related to the distance between the evidence signal generated from an item and the decision criterion it is judged against, with greater distances leading to faster responses (Koppell, 1977). Under the trial-by-trial criterion shifting account of sequential dependencies, the criterion will move towards the left of the distributions following an “old” response. This action will both increase the proportion of signals that cross the “old” response threshold (thus increasing the probability of giving an “old” response on the next trial) as well as create more distance between the criterion and signals on the right side of the distribution (and less distance from the left side of the distribution). This second feature should produce faster “old” responses and slower “new” responses following an “old” decision compared with a static criterion. Alternatively, under an evidence change account like the “sticky evidence” model from Kantner et al. (2019), when an “old” decision is made, the evidence signal on the subsequent trial will be pulled towards the previous signal. This will result in some signals crossing the criterion, thus increasing “old” perseveration. In addition, for some “old” signals being pulled towards a previous “old” decision, they will be pulled further away from the criterion, thus increasing the relative distance from the criterion and increasing the decision speed. The reverse patterns would be expected if the previous decision was “new.” Therefore, an account that assumes mnemonic contributions to sequential dependencies is compatible with both the increased probability of making a repeat decision and the increased speed with which repeat decisions are made.

A potential limitation of the current study is the use of nonword stimuli across experiments. In particular, readers may note the low rates of *d*′ across the experiments as a cause for concern. However, the *d*′ measured in this study are entirely in line with those measured in Dollois et al. (2024) when using nonword stimuli and thus not a surprise given our methods. In addition, as comparable decision perseveration is seen when using standard English words (Dollois et al., 2024) and pictures (Malmberg & Annis, 2012), we don’t have reason to believe that the results of the current experiment are substantially impacted by stimulus choice.

## Conclusion

Across three experiments, we have found consistent evidence that decisions perseverate during recognition tests. We found no convincing evidence that this perseveration is a product of motor response priming. That is to say, the tendency to repeat decisions at test does not appear to be the product of a tendency to repeat motor actions, as is showcased by its persistence even when motor priming is prevented. In addition, we found that decisions were executed faster when they repeated between trials compared with when they switched, even without the contribution of motor priming. Both behavioural patterns are consistent with accounts of sequential dependencies anchored on mnemonic changes between trials. This work highlights that response perseveration is a *decision*-based effect.
